# Complete Genome Sequence of a Lineage II Peste des Petits Ruminants Virus from Sierra Leone

**DOI:** 10.1128/genomeA.01417-17

**Published:** 2018-01-04

**Authors:** William G. Dundon, Caroline M. Adombi, Saidu Kanu, Angelika Loitsch, Giovanni Cattoli, Adama Diallo

**Affiliations:** a Joint FAO/IAEA Division of Nuclear Techniques in Food and Agriculture, Department of Nuclear Sciences and Applications, International Atomic Energy Agency, Vienna, Austria; b Institute of Agropastoral Management, University Peleforo Gon Coulibaly, Korhogo, Ivory Coast; c Department of Animal Science, Njala University, Freetown, Sierra Leone; d Institute for Veterinary Disease Control, Austrian Agency for Health and Food Safety, Mödling, Austria; e CIRAD, Biological Systems Department, UMR CIRAD-INRA Animal, Santé, Territoires, Risque et Environnement (ASTRE) and ISRA-LNERV, Dakar-Hann, Senegal

## Abstract

The complete genome sequence of a peste des petits ruminants virus (PPRV) from goat samples collected in Sierra Leone in 2011 is reported here. The genome shows a higher nucleotide sequence identity (98.9%) with a lineage II PPRV from Senegal than to PPRVs from neighboring Liberia and Ivory Coast.

## GENOME ANNOUNCEMENT

Sheep and goats contribute considerably to the cash income and nutrition of small farmers in many countries, so the control of peste des petits ruminants (PPR), a highly infectious transboundary viral disease of small ruminants, is considered an essential element in the fight for global food security and poverty alleviation (1). For this reason, PPR is presently being targeted by international organizations for global eradication by 2030 (2).

The causal agent of PPR is the PPR virus (PPRV), which is one of the members of the genus *Morbillivirus* within the family *Paramyxoviridae* (1). It is a nonsegmented, negative, and single-stranded RNA virus that encodes six structural proteins, nucleocapsid protein (N), phosphoprotein (P), matrix protein (M), fusion protein (F), hemagglutinin protein (HA), RNA-dependent RNA polymerase (L), and two nonstructural proteins (V and C). The virus has been classified into four genetic lineages based on the comparison of a sequence fragment from either the N or F gene (2). Lineage IV is prevalent in Asian countries, but since 2008, it has also been detected in many countries in Africa. Lineages I, II, and III have been found primarily in Africa (1).

In December 2011, in Moyamba, southwestern Sierra Leone (8°09′38″N 12°26′0″W), pathological and swab samples were collected from goats during a suspected PPR outbreak. The samples were transported on ice to Njala University, Freetown, Sierra Leone, and then shipped on dry ice to the Austrian Agency for Health and Food Safety for further characterization. RNA was extracted from the samples and analyzed by reverse transcription-PCR (RT-PCR) using primers for a segment of the PPRV N gene, as described previously (3). Phylogenetic analysis of the amplicons generated from positive tissue samples revealed that they contained viral RNA from a lineage II PPRV. The RNA from one positive lung sample (PPRV/Sierra Leone/048/2011, also known as Moyamba) was then selected for genome sequencing, as described previously (4).

The PPRV/Sierra Leone/048/2011 genome is 15,948 bp long. Like all PPRVs sequenced to date, there is a 107-nucleotide (nt) genome promoter region at the 3′ end, immediately followed by the transcription units for the N, P, M, F, H, and L proteins and the antigenome promoter at the 5′ end. The genome has the highest nucleotide sequence identity (98.9%) with a lineage II virus identified in Senegal in 2013 (GenBank accession number KM212177). Interestingly, the strain PPRV/Sierra Leone/048/2011 has a lower nucleotide sequence identity with PPRVs from neighboring countries, e.g., Liberia (accession number KU236379) (98.6%) and Ivory Coast (accession number KR781451) (98.6%) collected in 2009 and 2015, respectively. Whether this nucleotide difference indicates a common source of PPRV from Senegal and Sierra Leone and, as such, reflects the transboundary movement of PPRV in the region requires further in-depth molecular epidemiological studies at a regional level.

Although PPR in Sierra Leone has been reported (5), this is the first complete genome of a PPRV from the country, providing additional information on the circulation of this important virus in West Africa.

### Accession number(s).

The complete genome sequence of PPRV/Sierra Leone/048/2011 has been deposited in GenBank under the accession number MF741712.
